# Polyurethane Leather as a Suture Training Model

**DOI:** 10.7759/cureus.71182

**Published:** 2024-10-10

**Authors:** Panuwat Khantasa-Ard

**Affiliations:** 1 Department of Surgery, Mahasarakham Hospital, Mahasarakham, THA

**Keywords:** polyurethane leather, pul, suture simulator, suture training model, suture training pad

## Abstract

In the suture training of medical students at Mahasarakham Hospital Medical Education Center, pig skin and butyl rubber were used, but both materials presented several problems. Pig skin was difficult to preserve, becoming waxy, easily rotten, and hard in texture while butyl rubber was hard and sticky causing difficulties in suture practice. This study aimed to create a new suture training model to replace the original models. A suture training pad was made by attaching polyurethane leather (PUL) to a sponge pad with fabric glue. Then the pad was attached to a wooden board using double-sided adhesive foam tape, completing the suture training model. The suture training model can be used for various surgical skills training for medical students, including incision, suturing, and knotting. The time taken to make a pad and a model was 7 and 10 minutes, respectively, and the cost of making one suturing model was 13.55 bahts (0.41 USD). The new suture training model of PUL is cost-effective, easy to self-assemble, and eliminates the need for complex production, making it a suitable replacement for the original models.

## Introduction

Wounds are a condition frequently encountered in medical practice. There are many types of wounds, each requiring different treatments. Suturing is a common method used to promote wound healing. However, in specific situations such as active bleeding, large wounds, or wounds accompanied by vascular injury, doctors must act promptly and accurately to save the patient's life. Additionally, facial wounds require fine skills for reconstruction, making suturing an essential basic surgical procedure for doctors. All doctors must train in suturing to ensure effective, confident, and safe treatment for patients, leading to good results without complications [1].

All doctors are taught suturing from their time as medical students, beginning with training on simulators to develop their skills before performing on patients [2,3]. There are many suture training simulators available [4-13], each with different material characteristics and levels of fidelity [3].

At Mahasarakham Medical Education Center, Mahasarakham Hospital in Thailand, medical students previously trained on pigskin and butyl rubber for suturing. However, pigskin was difficult to preserve, becoming waxy, easily rotten, and quite hard, while butyl rubber was also problematic due to its hardness and stickiness, making suturing difficult. Although commercial silicone pads were easier to use and preserve, their high cost was a drawback. Therefore, the author, a general surgeon and medical lecturer at this institute, proposed creating a suture training model using easily accessible and inexpensive materials with a straightforward production process. The model is designed to be easy to use, reusable, efficient, and suitable for medical students, allowing them to practice suturing in private accommodations. This study aimed to describe a new suture training model made from polyurethane leather (PUL) as a replacement for the previous simulators.

## Technical report

**Figure 1:**
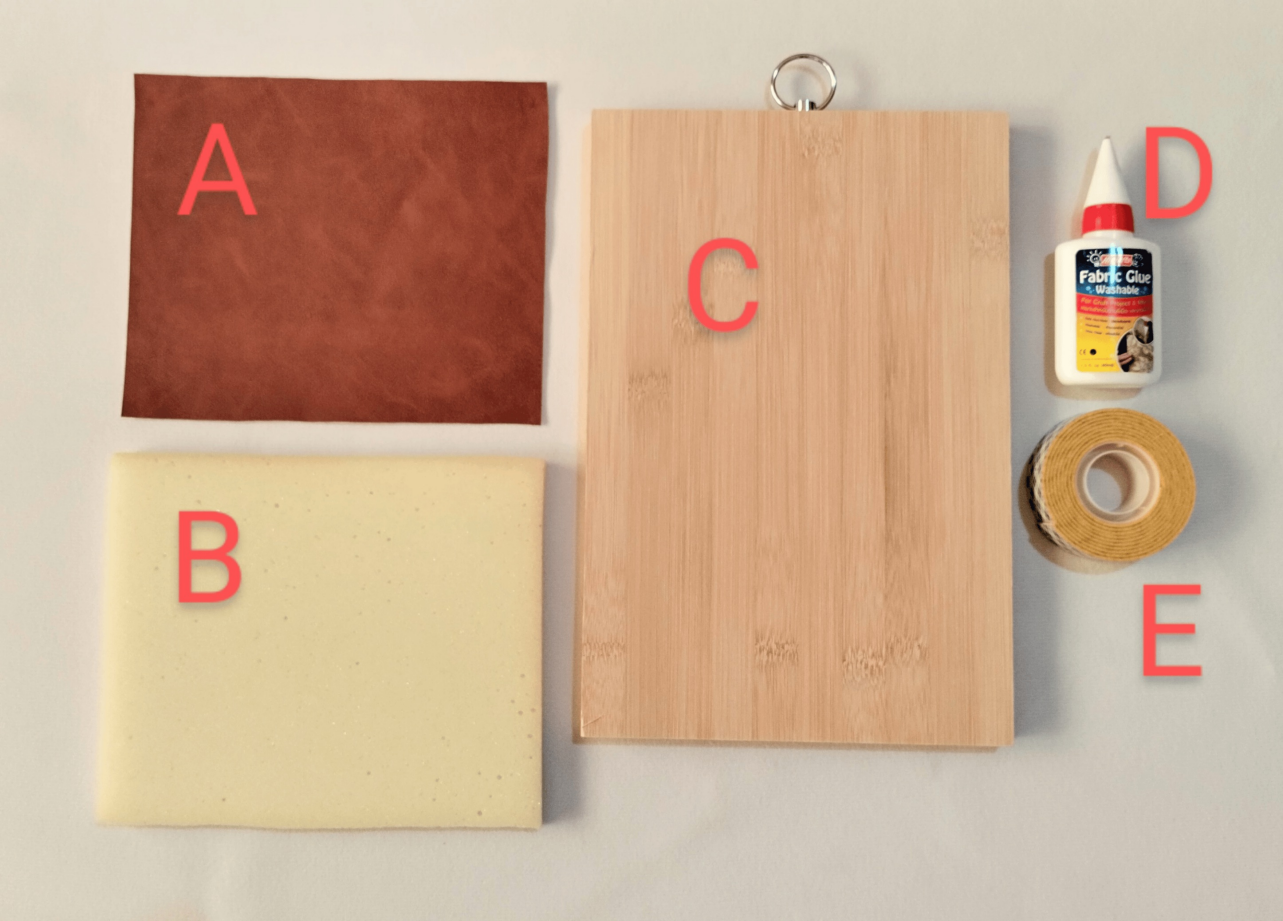
The materials for making the PUL suture training model A = PUL (Polyurethane Leather) plate; B = sponge pad; C = wooden cutting board; D = fabric glue; E = double-sided adhesive foam tape

A novel suture training model includes a suture training pad attached to a cradle. The materials for making the suture training pad are a plate of PUL (16 cm width, 19 cm length, and 1 mm thickness), a sponge pad (16 cm width, 19 cm length, and 1.5 cm thickness), and fabric glue. The materials for making the cradle are a wooden cutting board (18 cm width, 28 cm length, and 1.5 cm thickness) and double-sided adhesive foam tape (21 mm width) (Figure 1).

**Figure 2:**
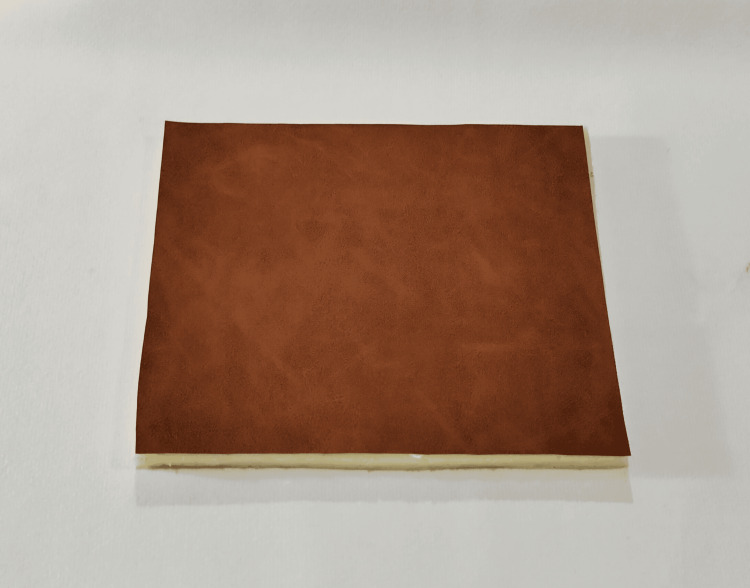
The PUL suture training pad PUL = Polyurethane Leather

**Figure 3:**
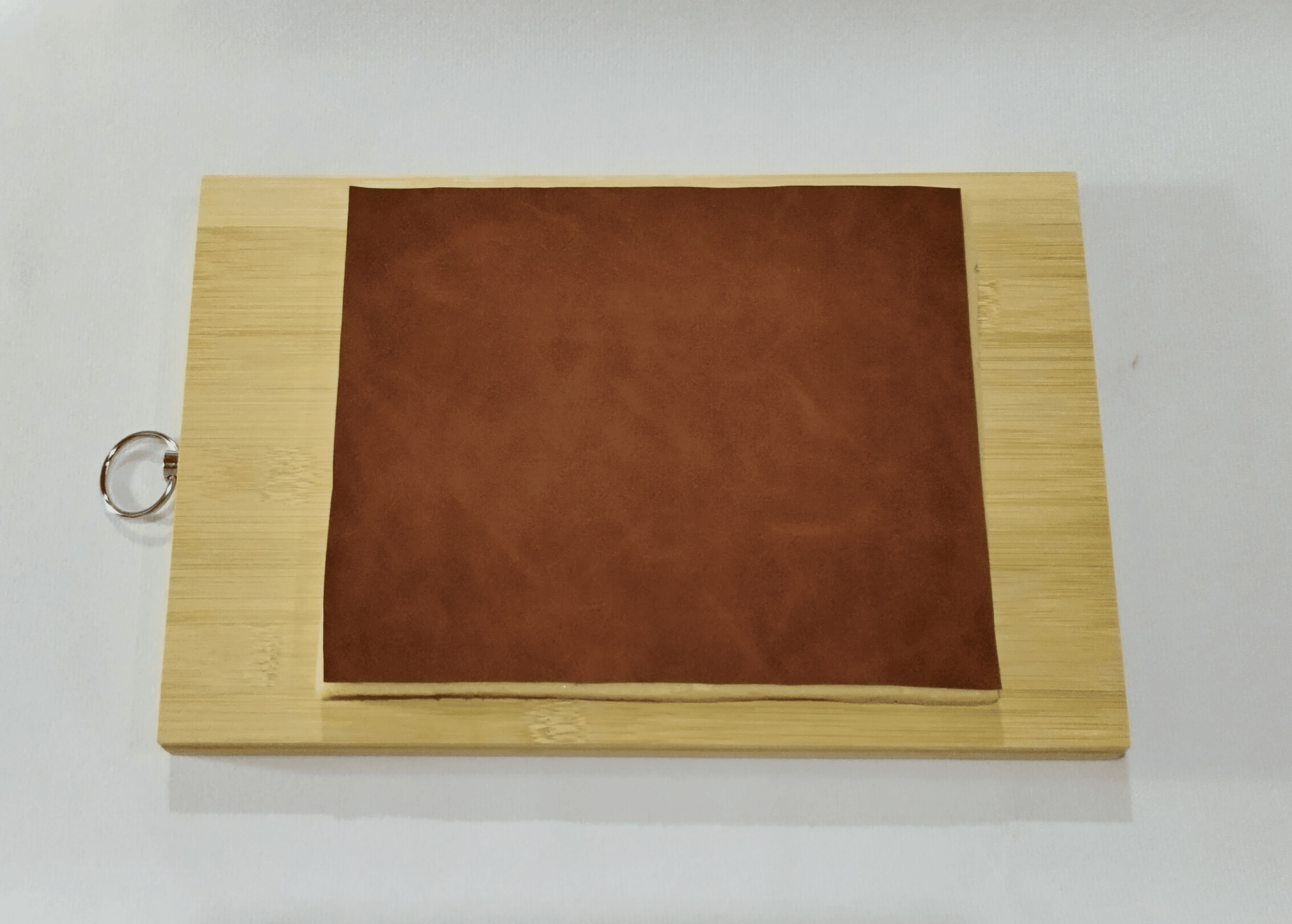
The PUL suture training model PUL = Polyurethane Leather

The suture training pad can be created by applying glue thoroughly on the reverse surface of the PUL. Then, place the sponge pad together with the PUL. Press the pad with a hand or a wooden board to ensure a firm attachment between the PUL and the sponge pad. Leave it at room temperature for 5 minutes to allow the glue to dry, completing the suture training pad (Figure 2). Next, thoroughly adhere small pieces of foam tape to the sponge side of the suture training pad, then attach the pad to a wooden cutting board to complete the suture training model (Figure 3).

The PUL suture training model can be used in the surgical skill training of medical students and includes the following:

Surgical Incision Training: Medical students can learn about handling the blade, using a scalpel, assembling the blade and scalpel, holding the scalpel, making the incision line, and managing the length, width, and depth of the incision.

**Figure 4:**
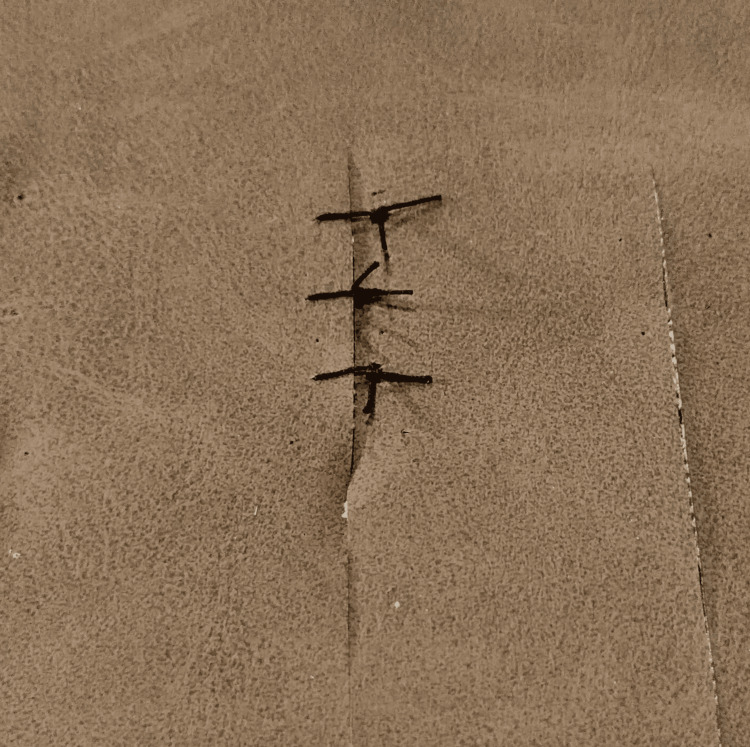
Simple interrupted suture on the PUL suture training model PUL = Polyurethane Leather

**Figure 5:**
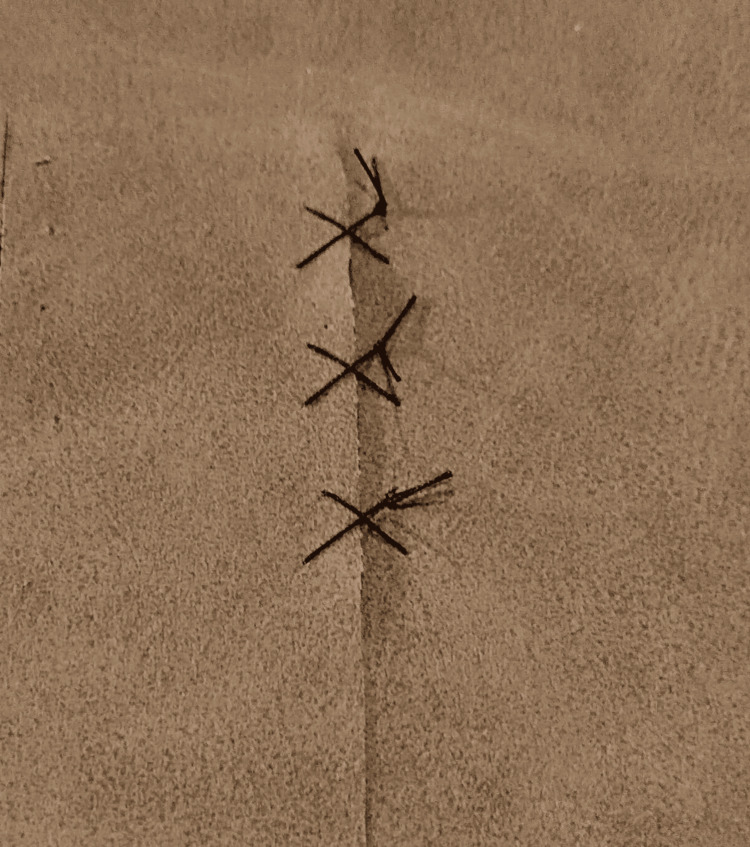
Figure of eight stitches on the PUL suture training model PUL = Polyurethane Leather

Suturing Training: Students begin by familiarizing themselves with suturing instruments, including the needle holder, forceps, scissors, and various types and sizes of sutures. They will practice instrument handling methods, using the model for various types of suturing (simple interrupted, vertical/horizontal mattress, continuous suture, and figure-of-eight) with both monofilament and multifilament sutures, and knotting with instruments (Figures 4, 5).

**Figure 6:**
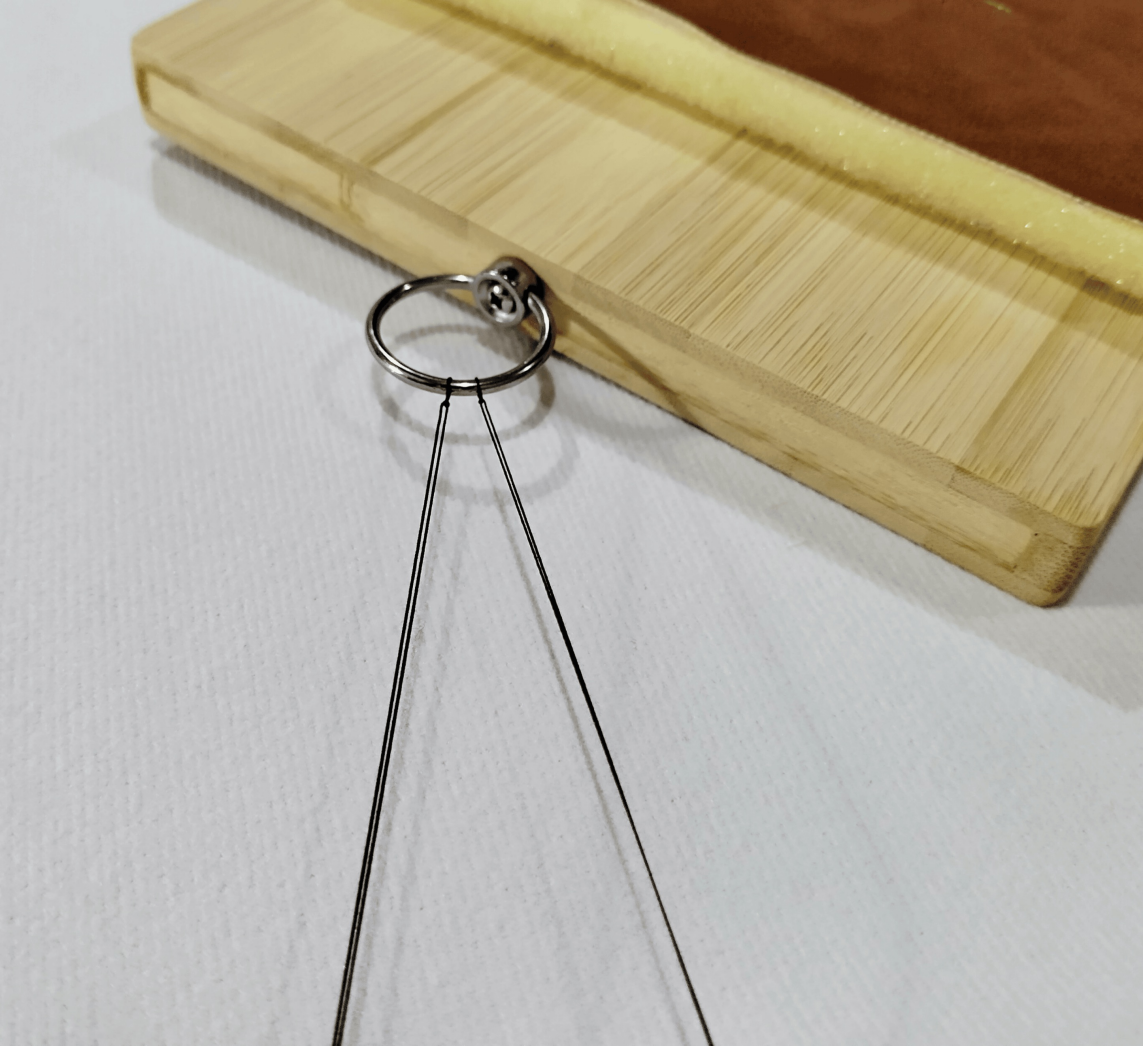
Knotting training with the metal loop beside the cradle

Knotting: The metal loop beside the cradle can be used for knotting training, employing both one- and two-hand techniques (Figure 6).

In the production process of a model, the time consumed is as follows: 2 minutes for applying glue on PUL, 5 minutes for the glue to dry (totaling 7 minutes for the suture training pad), then 2 minutes for adhering foam tapes, and 1 minute for attaching the pad to the wooden board. This sums up to a total of 10 minutes for the production of a model.

The overall cost of a suture training pad is 13.55 Bahts or 0.41 USD (32.92 Bahts = 1 USD, exchange rate as of September 23, 2024) [14]. This includes the following components: PUL at 4.69 Bahts (0.14 USD), sponge pad at 3.13 Bahts (0.1 USD), fabric glue at 5.29 Bahts (0.16 USD), and foam tape at 0.44 Bahts (0.01 USD).

The cost of the wooden board is 56 Bahts (1.7 USD). However, since the board is durable, reusable, and has a long service life, it is not included in the production cost of the pad.

## Discussion

A suture training model is a simulator that helps medical students progress their surgical skills through the learning curve, develop experience, and build confidence for practicing on real patients. This leads to smoother procedures, higher quality, fewer complications, and increased patient safety [1,2]. 

The ideal suture training model for surgical skill training should have a smooth surface, a soft and elastic texture similar to real human skin (fidelity), and be divided into layers (epidermis, dermis, subcutaneous tissue) like human skin. Many suture training models are produced and developed, both commercially and self-made. Self-made models address the high cost problem of commercial models [12]. Various studies have explored self-made models using synthetic materials like open-cell foam and elastic foam tape [2], silicone pads [5,11], Ethylene-Vinyl Acetate (EVA) [10], synthetic foam sponges [12], mouse pads [13], and living materials such as beef tongue [4], pig feet [5], chicken legs [6], banana peels [7,15], orange peels [8]. Each model varies in difficulty level and suitability for skill training.

This study presents a suture training model made from PUL, a synthetic leather used for wrapping sofas. PUL has a smooth surface, is soft and elastic, hard to tear but can be easily cut or punctured, making it suitable for a suture training model. The PUL is attached to a sponge pad to mimic human skin layers, with the PUL representing the epidermis and dermis, and the sponge pad representing subcutaneous tissue, as suggested by Janus et al. [2] and Yag-Howard [13]. Fabric glue was found to be easier to use than other adhesives and provided a strong bond between the reverse side of the PUL and the sponge pad. Attaching the suture training pad to a wooden board increased the weight of the model, preventing movement during skill training and ensuring smoother procedures, consistent with the concept of Antonopoulos et al [11].

The suture training model can be used for various types of suturing, especially basic techniques such as simple interrupted, mattress suture, and simple continuous suture, consistent with other studies [7,10]. It is compatible with both monofilament and multifilament sutures. Additionally, the model can be used for other surgical skills training, including surgical incisions, which are easy to create, and for practicing the direction and depth of incisions, as well as knotting training using the metal loop of the cradle.

However, the PUL suture training model has a limitation: the sponge pad is less tear-resistant and less elastic, making subcuticular or subcutaneous suturing difficult. The sponge layer cuts through when tension from the suture is applied.

The PUL suture training model can be used for advanced surgical technique training for medical students, including procedures like excision of skin lesions, flap coverage (e.g., local advancement flap, V-Y flap), and incision and drainage of cutaneous abscesses. Additionally, it can be enhanced for surgical resident skill training by using a box cover on the suture training pad for deep-site suture practice or by increasing the sponge thickness for retention suture training.

During the initial training period, a marker indicating the gap from the wound edge or between stitches could be used to help students become familiar with using their eyes in coordination with their hands to estimate the suturing gap.

Further studies should be conducted, such as evaluating medical students' experience with training on the suture training pad and the quality of their stitches, comparing the PUL suture training pad with other types of suture training pads, and improving the elasticity of the sponge layer for subcuticular and subcutaneous suture training.

## Conclusions

The PUL suture training model is made from easily obtainable and low-cost materials, requires no complicated production, and can be produced quickly. It is reusable, allows for pad replacement, and can be made by medical students themselves, enabling private practice. Additionally, it can be used for various basic surgical skill training. Therefore, the PUL suture training model is an excellent and efficient choice for medical students, capable of replacing the original models.
